# Comperative evaluation of the current and new design miniplate fixation techniques of the advanced sagittal split ramus osteotomy using three-dimensional finite element analysis

**DOI:** 10.4317/medoral.25964

**Published:** 2023-10-12

**Authors:** Fuat Altuncu, Dilara Kazan, Bora Özden

**Affiliations:** 1 Private Dental Clinic, Samsun, Türkiye; 2 Bahcesehir University, Faculty of Dentistry, Department of Oral and Maxillofacial Surgery, İstanbul, Türkiye

## Abstract

**Background:**

The aim of this study was to evaluate the stress occurring in the fixation systems both developed in various geometric designs for this study and currently used in sagittal split ramus advancement osteotomy using finite element analysis.

**Material and Methods:**

The finite element model that imitates three-dimensional sagittal split advancement osteotomy was fixed in 10 different miniplate fixation methods: one miniplate fixed with four monocortical screws in a horizontal and oblique pattern; four-hole two miniplates with eight monocortical screws; five-hole miniplate fixed with four monocortical and one bicortical screws; six-hole straight and curved miniplates fixed with six monocortical screws in different geometric designs. Unilateral masticatory muscle loads that have previously determined in the literature were applied to the model at the anatomical muscle attachment regions and the data obtained from finite element analysis and static linear analysis methods were recorded as Von mises, maximum principle and minimum principle stress values.

**Results:**

It was observed from the results that maximum stress occurred in Group 1, which consisted of double backward T-shaped miniplate with 6-holes and, minimum stress occured in group 10, which mimiced hybrid system with one miniplate and four monocortical and one bicortical screws.

**Conclusions:**

Based on our results, the stress on the miniplates changes according to the geometric designs and the stress on the miniplate decreases as the numbers of miniplates and bars increase. The hybrid miniplate may be preferred by the surgeon as it will be exposed to less stress in excessive mandibular advancements by using the advantages of both the miniplate and the bicortical screw.

** Key words:**Sagittal split ramus osteotomy, biomechanics, miniplate, finite element analysis, fixation methods.

## Introduction

Bilateral Sagittal Split Ramus Osteotomy (SSRO) is the most commonly used surgical procedure in orthognathic surgery for correction of mandibular deformities, as it allows 3D corrections of the mandible (1). Providing adequate stability after orthognathic surgery is important to minimize complications and relapse. Particularly in cases where the mandible is advanced, segments need more sTable fixation systems because the bone contact between the segments are reduced, the tissues adjacent to the segments are stretched, and tend to displace the distal segment back toward its original poisition (2).

Today, the use of rigid internal fixation systems has enabled us to obtain more predicTable successful results than wire osteosynthesis systems (3). Considering the advantages and disadvantages, three basic fixing methods have been used that have proven their success so far: bicortical screws, miniplates with monocortical screws and hybrids methods. Biomechanical studies investigating the properties of various fixation techniques in mandibular osteotomies include in-vitro and finite element analysis studies (4,5). The finite element method allows the analysis of biomechanical properties of bone and fixation techniques in various situations and loads.

There are many reports in the literature of *in vitro* analysis of osteotomy stabilization using 2.0-mm bicortical screw fixation in various patterns. Traditionally, the use of three interpositional bicortical screws placed in an "inverted-L" pattern has been considered the "gold standard" for having the best mechanical behavior in mandibular advancements (6). However, using bicortical screws method carries a risk of damage to adjacent anatomical structures such as teeth, nerves or vessels, due to the need for transcutaneous access.

Another rigid internal fixation method is miniplate fixation with monocortical screws. The main advantage of miniplate fixation is that the monocortical screws passes only through the buccal cortex so that does not cause any nerve damage and there is no need to use a buccal trocar compared to bicortical screw fixation. Although miniplate fixation does not provide as rigid biomechanical resistance as bicortical screws, it is one of the best alternatives for great advanced mandible mobilization needs. Moreover, it can be applied more than once to increase the biomechanical resistance against chewing forces. Considering these advantages, miniplate fixation has become the most preferred method among surgeons (7,8).

Mini-plates are suiTable to be modified both in designs such as straight, curved, Y, Z, Square or L-shaped, and in different geometrical arrangements such as oblique or straight, upon use of two parallel mini-plates or hybrid forms by placing them on the midline, lower or upper edge of the osteotomy line.

Although clinical evidence indicates that one miniplate is sufficiently sTable in short advancements (9-12), application of a single miniplate in large mandibular advancements (10 mm or more) may be risky due to the excessive chewing forces to which the segments will be exposed (8). In such cases, stability can usually be achieved with an additional bicortical screw or an additional miniplate to the posterior segment of the mandible (13). The hybrid technique combines both the advantages of the bicortical screws technique and the advantages of the miniplates and monocortical screws technique. Adding an additional bicortical screw/screws to the miniplate with monocortical screws fixation increases stability.

Although the sagittal split osteotomy technique has undergone various modifications to date, it is now a routine standard procedure and its fixation is provided with rigid internal fixation systems. However, which internal fixation system provides the best fixation remains unclear. In addition, as evaluated in this study, the effect of the increase in the number of mini-plates, its inclination, and the geometric design of the monocortical screws used with it on stabilization have not been fully revealed.

Since controversial results have been obtained as a result of many biomechanical test studies or finite element analysis studies on the stability of miniplate fixations to date (14-16), research continues on whether a special mini-plate to be produced can provide more stability.

In this presented study, it was aimed to verify the stress and displacement by finite element analysis (FEA) of ten different types of rigid internal fixation and to compare the stability and stress of conventional, new designed and custom-mini plate systems. It was tried to select groups that are considered to be the most sTable by the authorities and whose stability can be increased by modification. It was also sought to answer the question of whether stability is increased when a miniplate design that mimics the geometric design of the bicortical screw is used, as bicortical screws are known to be more rigid.

## Material and Methods

This research was conducted through colloboration between Samsun Ondokuz Mayıs University Faculty of Dentistry and Ay Tasarım Ltd. For this study, computer system was used that had Intel Pentium®D CPU 3,00 GHz processor(2200 Mission College Blvd,Santa Clara,CA 95054-1549 USA),Windows XP Professional Version 2002 Service Pack(39900 Corporate Campus Dr.,Suite 3000 Louisville Kentucky,40223 USA)operating system software, Rhinoceros 4.0(3670 Woodland Park Ave,Seattle,Washington, 98103 USA)modelling softwareand FEM Pro(Algor, Inc.150 Beta Drive Pittsburgh,Pennsylvania,15238-2932 USA)analysing sofware.

- Modelling

This study was carried out by using 3D finite element model analysis of human mandible. Hemimandible models were generated by 3D complex processing and transferred to VRMesh software for 3D mesh modelling. In Solidworks, both the cortical mandible layer and cancellous bone were combined to develop a corticocansellous mandible model. Generated bi-layer solid models underwent a virtual sagittal split osteotomy (Obwegeser technique with DalPont and Hunsuck modifications), which resulted in 2 segments of the mandible: proximal and distal. A 10 mm advancement of SSRO was simulated in a 3D mesh model of mandible according to the DalPont-Hunsuck modifications.

- CAD Modelling of Hardware

Demo models of screws and miniplates supplied in the study were scanned in 3D on a macro scale with smartoptics 3D scanner. Models obtained in the .stl format were sent to Rhinoceros 4.0(3670 Woodland Park Ave N,Seattle, WA 98103 USA). In rhino software, the Boolean method was harmonization between the miniplate and the bone, screws and bone and miniplate. In this way, the mandible, temporomandibular joint and disc, miniplates and mono and bicortical screws were moved to the model to reflect its true morphology. All monocortical screws with a diameter of 2 mm and a length of 5 mm. The bicortical screw for Group 10 is modeled as 12 mm lenght.

Young’s moduli of cortical bone, spongious bone, and titanium(Ti-6Al-4V) were 14.8 GPa, 1.85 GPa, and 110 Gpa, respectively. Poisson’s ratio used in the analysis was 0.3 for bone and 0.33 for titanium alloy (17).

- Creation of Experimental Groups

**Figure 1:**
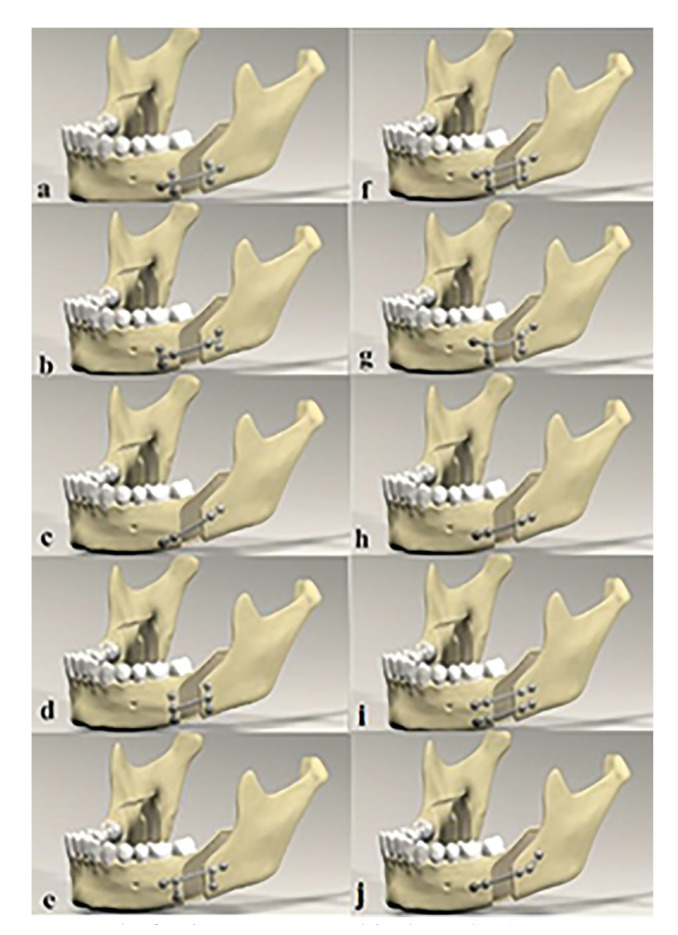
The fixation groups created in the study. a) Group 1: One double backward T-shaped miniplate with 6-holes. b) Group 2: One double T-shaped miniplate with 6-holes. c) Group 3: One four-hole straight miniplate placed obliquelly. d) Group 4: One six-hole miniplate with 3 screws in vertical form to each other. e) Group 5: One six-hole straight miniplate formed by combining 2 inverted-L-shaped plates with a bar. f) Group 6: The same design as Group 5, with two bars. g) Group 7: The same design as Group 5, with curved miniplate. h) Group 8: One four-hole straight miniplate placed horizontally. i) Group 9: Two four-hole miniplate placed horizontally. j) Group 10: Hybrid miniplate.

In this study, 10 group of different miniplate fixation methods were created for advancement of Sagittal Split Osteotomy (Fig. 1). All screws to be applied were 2mm diameter and 5mm long monocortical screws. Only for Group 10, the most distal screw was designed as a 12mm bicortically.

These ten groups were selected from the techniques previously proven to be highly sTable in the literature and their modifications designed for this study, considering that these techniques would increase stability.

These modifications were made by:

1. changing the geometric patterns of monocortical screws, as in bicortical screws (3 screws in T-pattern, backward T-pattern, vertical form and inverted-L form)

2. increasing the number of bars (one/two)

3. changing the curve (straight/curved)

4. changing the placement (horizontal/oblique)

5. creating a hybrid technique by adding a bicortical screw, in order to examine the effects of many parameters on stability.

Group 1: One six-hole double backward T-shaped miniplate-fixed with 6 monocortical screws (Fig. 1).

Group 2: One six-hole double T-shaped miniplate-fixed with 6 monocortical screws (Fig. 1).

Group 3; One four-hole straight miniplate placed obliquely-fixed with 4 monocortical screws (Fig. 1).

Group 4: One six-hole miniplate, with 3 holes vertically in each side unioned with a bar-fixed with 6 monocortical screws (Fig. 1).

Group 5: One six-hole straight miniplate formed by combining 2 inverted-L-shaped plates with a bar-fixed with 6 monocortical screws (Fig. 1).

Group 6: The same design as Group 5, with two bars-fixed with 6 monocortical screws (Fig. 1).

Group 7: The same design as Group 5, with curved miniplate-fixed with 6 monocortical screws (Fig. 1).

Group 8: One four-hole straight miniplate placed horizontally-fixed with 4 monocortical screws (Fig. 1).

Group 9: Two four-hole miniplate placed horizontally-fixed with 8 monocortical screws (Fig. 1).

Group 10: Hybrid miniplate (Five-hole obliquely placed miniplate-fixed with four monocortical screws, and one bicortical screw on most distally) (Fig. 1).

- Transferring Three-Dimensional Models to Finite Element Analysis

After the models were created geometrically with the VRMesh software, they were transferred to Algor Fempro (Algor Inc., USA) software in .stl format to be ready for analysis. The material (modulus of elasticity and Posison ratio) values ​​describing their physical properties are given to each of the structures that make up the models (Table 1).

**Table 1:**
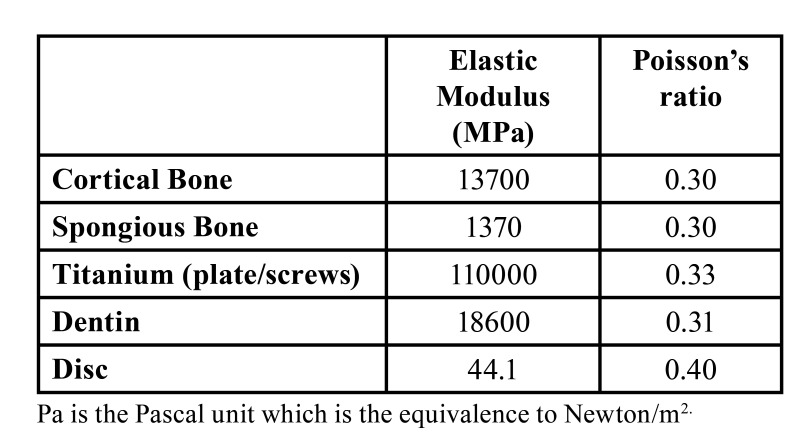
Basic physical properties.

Models made in VR Mesh software were imported into Algor software as surface data in the form of .stl. In order to be able to analyze in Algor software, the meshing process was performed in a solid form.

The number of elements and nodes used in the mathematical models containing the scenarios are given in Table 2.

In the software program, solid body properties were accepted as linear elastic, homogeneous, and isotropic. Since the values obtained as a result of FEA were the results of mathematical calculations without variance, no statistical analyses could be made.

**Table 2:**
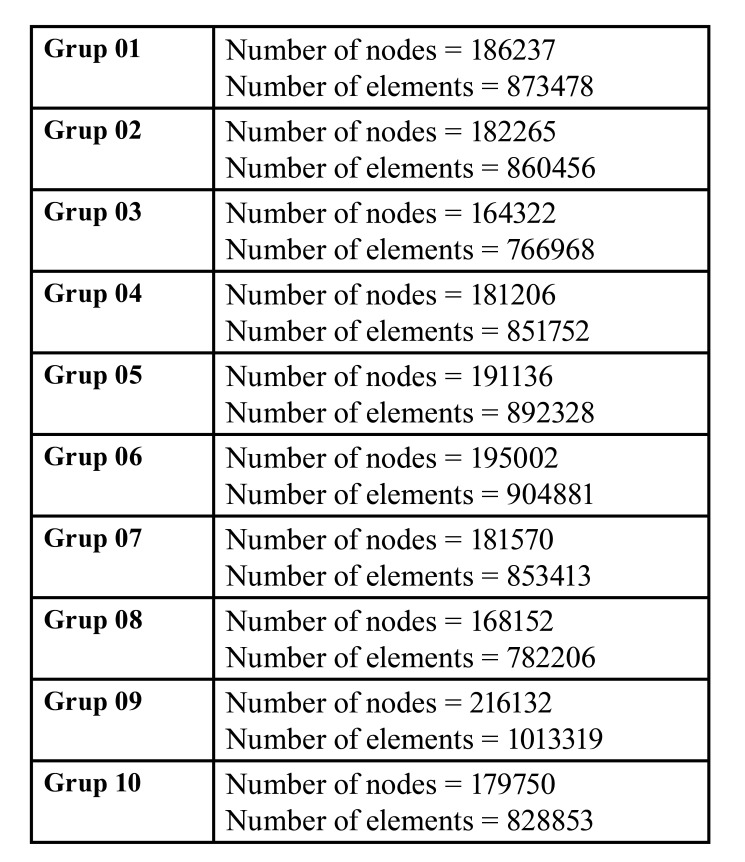
Number of nodes and elements in the experimental groups.

- Loading and Boundary Conditions

All the models were fixed from the glenoid fossa of the temporal bone to have 0 degree of freedom (DOF) motion. In addition, X symmetry is given from the middle axis of the model, that is, from the medial line. In addition, the buccal direction was limited in a certain area in the proximal part to represent the cheek muscles.

**Figure 2:**
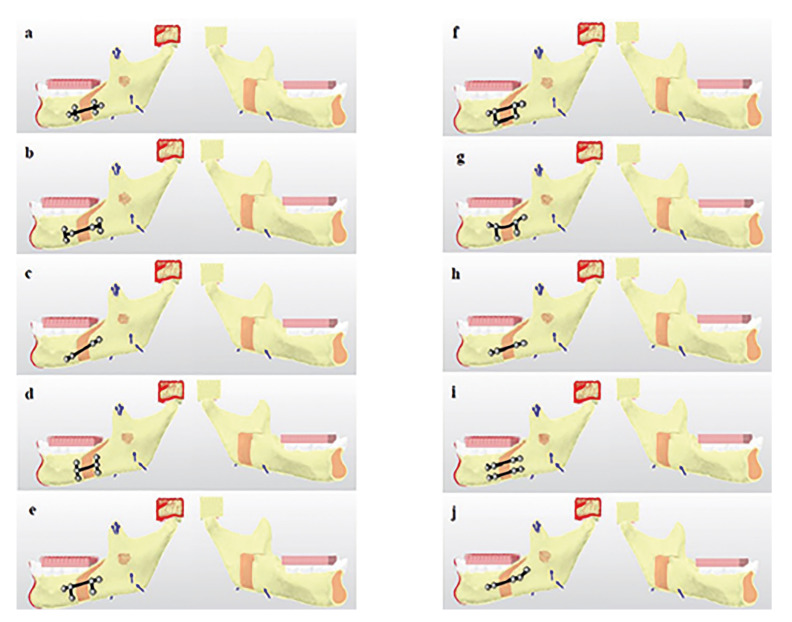
Directions of application of muscle forces and boundary conditions. (One arrow: medial pterygoid muscle, two arrows: masseter muscle, and three arrows: temporal muscle). a) Group 1: One double backward T-shaped miniplate with 6-holes. b) Group 2: One double T-shaped miniplate with 6-holes. c)Group 3: One four-hole straight miniplate placed obliquelly. d) Group 4: One six-hole miniplate with 3 screws in vertical form to each other. e) Group 5: One six-hole straight miniplate formed by combining 2 inverted-L-shaped plates with a bar. f) Group 6: The same design as Group 5, with two bars. g) Group 7: The same design as Group 5, with curved miniplate. h) Group 8: One four-hole straight miniplate placed horizontally. i) Group 9: Two four-hole miniplate placed horizontally. j) Group 10: Hybrid miniplate.

In the light of anatomical data, using the values referenced by many studies in this field, anatomical force vectors were placed in the regions where the masticatory muscles attach to the mandible in the 3D model, and the relevant force magnitudes imitating unilateral biting forces were assigned to these vectors (Fig. 2), (Table 3).

**Table 3:**
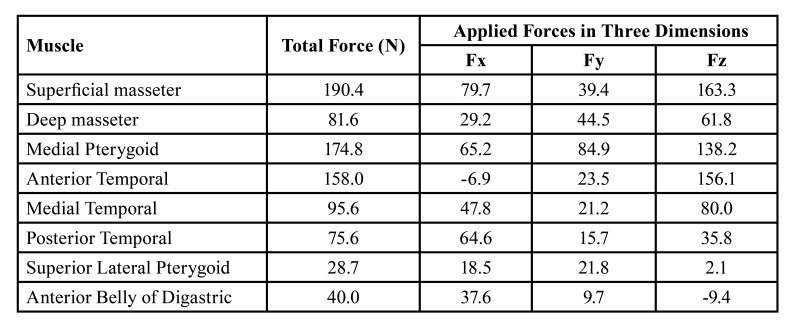
Forces of occlusion exerted by the muscles applied onto the mandible in the finite element analysis.

## Results

- Stress Values on Miniplates

At 10mm advancement, the magnitude of the stress on the plates was found to be significantly different between the groups. As a result of this finite element study, remarkably lower stress values were observed on the hybrid-miniplate group when compared with the other miniplate groups. The highest stress among the groups was recorded in Group 1 (558.82N/mm2), where 2 vertical screws were placed close to the osteotomy line and one screw was placed away from the osteotomy line, and the lowest stress value was found in Group 10 (128.27N/mm2) (Table 4).

**Table 4:**
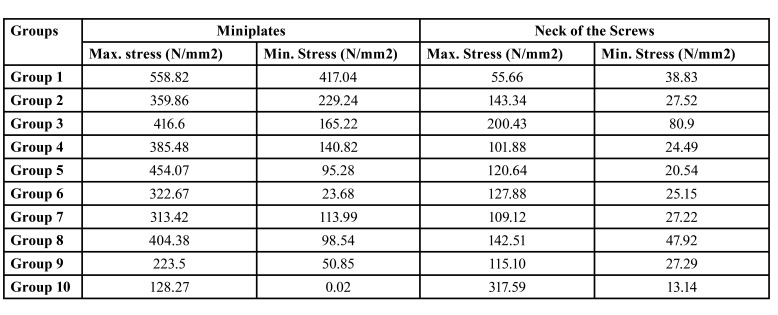
Maximum and minimum Von Mises stress values created on the miniplates and neck of the screws in different groups.

**Figure 3:**
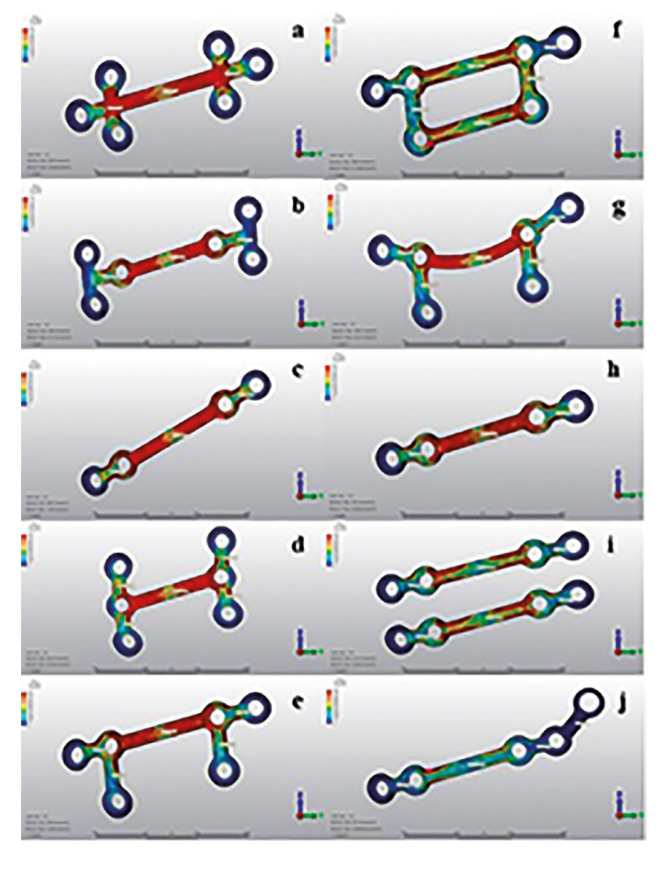
Stress values ​​on miniplates in all groups. a) Group 1: One double backward T-shaped miniplate with 6-holes. b) Group 2: One double T-shaped miniplate with 6-holes. c) Group 3: One four-hole straight miniplate placed obliquelly. d) Group 4: One six-hole miniplate with 3 screws in vertical form to each other. e) Group 5: One six-hole straight miniplate formed by combining 2 inverted-L-shaped plates with a bar. f) Group 6: The same design as Group 5, with two bars. g) Group 7: The same design as Group 5, with curved miniplate. h) Group 8: One four-hole straight miniplate placed horizontally. i) Group 9: Two four-hole miniplate placed horizontally. j) Group 10: Hybrid miniplate.

In all of the miniplates, it was observed that the stress was concentrated on the bars close to the screws (Fig. 3).

- Stress Values on Neck of Screws

Von Mises stresses in the neck regions of the screws that fit into the plate threads were examined for each group.

**Figure 4:**
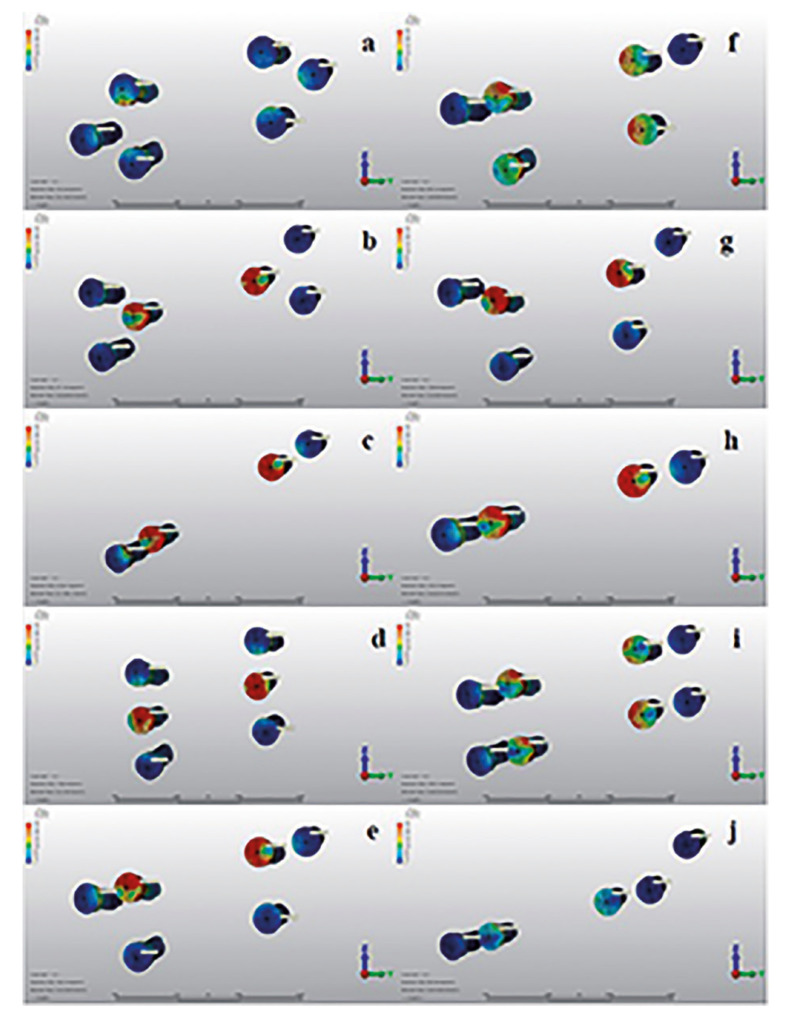
Stress distribution on the neck region of screws in all groups. a) Group 1: One double backward T-shaped miniplate with 6-holes. b) Group 2: One double T-shaped miniplate with 6-holes. c) Group 3: One four-hole straight miniplate placed obliquelly. d) Group 4: One six-hole miniplate with 3 screws in vertical form to each other. e) Group 5: One six-hole straight miniplate formed by combining 2 inverted-L-shaped plates with a bar. f) Group 6: The same design as Group 5, with two bars. g) Group 7: The same design as Group 5, with curved miniplate. h) Group 8: One four-hole straight miniplate placed horizontally. i) Group 9: Two four-hole miniplate placed horizontally. j) Group 10: Hybrid miniplate.

It was recorded that the highest stress value on the neck of screws belonged to the posterior bicortical screw in Group 10 (317.59N/mm2). It was also observed that all of the monocortical screws in Group 10 were exposed to less stress than the screws in the other group. When the monocortical screws in other groups were evaluated, it was observed that the highest stress value was observed in Group 3 (200.43N/mm2), while the stress values observed in Group 1 were remarkably low (55.66N/mm2). In general, the highest stress values on the neck of the screws were found to occur on the screws in the distal segment (Fig. 4).

- Stress Values on Cortical and Spongious Bone

The maximum stress recorded for the bone was observed in the region close to the screw in all groups. While the lowest stress value on cortical bone was found in Group 10 (41.58N/mm2), the highest stress value on sponigous bone was also found in the same group (36.70N/mm2).

## Discussion

SSRO is considered the standard surgery to correct mandibular insufficiency (18). The major concern in this osteotomy technique is postoperative relapse, which is unpredicTable and has a multifactorial etiology. Some of the factors contributing to relapse include inadequate fixation periods or method of fixation, magnitude of distal segment advancement, condylar malposition in the glenoid fossa, posterior migration in response to soft tissue and muscle pull, lack of control of the proximal segment during surgery.

Early relapse is usually caused by movement at the osteotomy site. Inadequate or improper fixation causes segmental movement due to altered muscle orientation. Another factor for early relapse is thought to be the amount of forward displacement of the distal segment (19-21). Kobayashi *et al*. (22) reported that advancement of greater than 10 mm increase tendency to relapse. If these two factors occur in the same patient, relapse will inevitably occur.

Today, miniplates, bicortical screws or a combination of the two systems(hybrid) have become the most commonly used techniques among rigid internal fixation techniques (23). Each fixation technique has its own advantages and advocates, and its application mostly depends on professional experience and preference.

Biomechanical tests and finite element analysis (FEA) are two main methods that have been frequently used to define the best fixation system in SSRO. FEA is a number-based method developed for use in engineering, and later used in dentistry to solve complex mechanical problems, and provides a close approximation of the *in vivo* geometry. Model detail verification and convergence studies are possible if the model is modeled in surface modeling tools (Surface First Approach) as parametric modeling. Since the model is parametric, trying multiple surface mesh detail leveling is possible to reach a convergence point. However, we have used mesh surface modeling (Mesh First Approach) in this study to get highly detailed and realistic organic 3d models that cannot be achieved by parametric surface modeling. The software we have used can import the mesh models (.stl files) and perform solid modeling and analysis. In this way, we gain the advantage of working on highly realistic 3d models without losing the ability to find the convergence point. However, since our models are highly detailed and the number of meshed nodes is far beyond any possible convergence point, we assume that we get rid of that disadvantage of the Mesh First Approach method.

Bicortical screw applications in different conFigurations were compared in the studies. The conFigurations consisted of 1 bicortical screw, 2 bicortical screws in a vertical pattern, 2 bicortical screws in a linear pattern, 3 bicortical screws in an inverted backward-L pattern, 3 bicortical screws in an inverted-L pattern and it was concluded that the 3 bicortical screws placed in inverted-L conFiguration had the highest stability (14,17,24,25). Despite the biomechanical benefits, the use of bicortical screws has serious biological disadvantages such as the possibility of condylar torque, risk of inferior alveolar nerve damage, difficulty in trocar insertion and the need for extraoral incision, and difficulty in removing the screws in case of infection or other complication (25,26). The present study is limited to miniplate geometric designs and a bicortical screw was used only to increase stability. However, the geometric designs of bicortical screws have been tried to be imitated with monocortical screws on the miniplate.

Another fixation method used after sagittal split ramus osteotomy is the use of monocortical screws and plates described by Michelet *et al*. in 1973 (27). This method is frequently used in SSRO due to its advantages such as intraorally placement of mini screws without the need for a transbuccal approach, less risk of inferior alveolar nerve damage, allowing function in the early postoperative period, minimal torsion at the condyle, and removal of the plate and screw under local anesthesia if necessary. In this present study, it was aimed to compare the popularly used miniplate fixation systems with the miniplates in different geometric conFigurations designed for this study.

Recently, many fixation studies have been reported in the literature comparing the stability of different miniplate and bicortical screw in different conFigurations. As a result of these studies, no significant differences that could affect stability were found. However, excessive mandibular advancements (more than 10 mm) require more rigid fixation. In this study, which we conducted to compare the stability of mini-plates of different designs, we also wanted to evaluate whether transferring the geometric design of the inverted L-shaped fixation with 3 bicortical screws, which is considered the most resistant fixation method in the literature, to the mini-plate will increase the stability. Group 5,6 and 7 represented this conFiguration and the maximum von Mises stress value was found in a six-hole straight miniplate formed by combining 2 inverted-L-shaped plates with a bar. However, a significant reduction in stress value was observed when an additional bar was added to the mini-plate or when a curved mini-plate was used.

Hybrid technique was described to take advantage of both the bicortical screw and monocortical screw-plate systems (28). In addition to a miniplate and four monocortical screws, hybrid fixation with a bicortical screw placed posterior to the last hole above the upper border of the inferior alveolar nerve increases stability. According to the results of our study, it was observed that the amount of stress in the 5-hole miniplate fixation method, in which the most distal obliquely placed screw was placed bicortically, was significantly lower compared to the other methods. This result shows that the addition of a bicortical screw to the miniplate significantly reduces stress. When one and two miniplates were compared, it was observed that the second miniplate significantly reduced the stress. Additionally, placing the miniplate obliquely rather than horizontally has been shown to reduce stress.

It has been reported in the literature that the screw closest to the osteotomy in the proximal segment is exposed to the highest stress in the system, followed by the screw closest to the osteotomy in the distal segment. In our study, it was observed that stress developed more in the screws closest to the osteotomy lines in all groups. This result is compatible with the literature. Moreover, considering that the stress will be higher in the regions close to the osteotomy lines, increasing the number of screws in this direction was not effective in reducing the stress.

As a result, if a clinical relevance is to be established with this finite element study, it has been determined that obliquely located 5-hole miniplate with four monocortical and one bicortical screws is exposed to less and homogeneous stress against chewing forces, and the resulting stress is concentrated in and around the bicortical screw. If a rigid fixation is required, choosing the last screw as bicortical in miniplates, increasing the number of bars or increasing the number of miniplates are alternative options that should be kept in mind.

There are some limitations to this FEA study. The study was limited to miniplates only and the bicortical screw was added to only one group with the thought that it would increase stability. Condylar torque that may occur after fixation has been disregarded. The models did not include the soft tissues of the temporomandibular joint (TMJ) and the condyle is fixed throughout the simulation. As in other FEA studies, the study was performed only on the mandible, ignoring the biomechanical effect on the maxilla and its soft tissues. We believe that clinical studies should be conducted to evaluate the effects of adapting the use of bicortical screws to miniplates of different designs on the stress formation on the TMJ, maxilla and soft tissues.

## References

[1] Watzke IM, Turvey TA, Phillips C, Proffit WR (1990). Stability of mandibular advancement after sagittal osteotomy with screw or wire fixation: a comparative study. J Oral Maxillofac Surg.

[2] Albougha S, Darwich K, Darwich MA, Albogha MH (2015). Assessment of sagittal split ramus osteotomy rigid internal fixation techniques using a finite element method. Int J Oral Maxillofac Surg.

[3] Turvey TA, Bell RB, Tejera TJ, Proffit WR (2002). The use of self-reinforced biodegradable bone plates and screws in orthognathic surgery. J Oral Maxillofac Surg.

[4] Tamura N, Takaki T, Takano N, Shibahara T (2018). Three-dimensional Finite Element Analysis of Bone Fixation in Bilateral Sagittal Split Ramus Osteotomy Using Individual Models. Bull Tokyo Dent Coll.

[5] Albougha S, Albogha MH, Darwich MA, Darwich K (2015). Evaluation of the rigidity of sagittal split ramus osteotomy fixation using four designs of biodegradable and titanium plates--a numerical study. Oral Maxillofac Surg.

[6] Foley WL, Frost DE, Paulin Jr WB, Tucker MR (1989). Internal screw fixation: comparison of placement pattern and rigidity. J Oral Maxillofac Surg.

[7] Sato FR, Asprino L, Fernandes Moreira RW, de Moraes M (2014). Comparison of postoperative stability of three rigid internal fixation techniques after sagittal split ramus osteotomy for mandibular advancement. J Craniomaxillofac Surg.

[8] Joss CU, Vassalli IM (2009). Stability after bilateral sagittal split osteotomy advancement surgery with rigid internal fixation: a systematic review. J Oral Maxillofac Surg.

[9] Özden B, Alkan A, Arici S, Erdem E (2006). In vitro comparison of biomechanical characteristics of sagittal split osteotomy fixation techniques. Int J Oral Maxillofac Surg.

[10] Sato FRL, Asprino L, Consani S, de Moraes M (2010). Comparative biomechanical and photoelastic evaluation of different fixation techniques of sagittal split ramus osteotomy in mandibular advancement. J Oral Maxillofac Surg.

[11] Olivera LBD, Manzato AJ, Guerra FLB, Arnett GW (2012). Biomechanical in vitro evaluation of three stable internal fixation techniques used in sagittal osteotomy of the mandibular ramus:a study in sheep mandibles. J Appl Oral Sci.

[12] Epker BN (1977). Modifications in the sagittal osteotomy of the mandible. J oral surg.

[13] Sigua-Rodriguez EA, de Medeiros RC, Goulart DR, Bomfim-Azevedo VL, Olate S, de Albergaria-Barbosa JR (2018). Comparative evaluation of different fixation techniques of the sagittal split ramus osteotomy in 10 mm advancements:mechanical testing and screw insertion torque. J Craniomaxillofac Surg.

[14] Stringhini DJ, Sommerfeld R, Uetanabaro LC, Leonardi DP, Araújo MR, Rebellato NLB (2016). Resistance and stress finite element analysis of different types of fixation for mandibular orthognathic surgery. Braz Dent J.

[15] Sigua-Rodriguez EA, Caldas RA, Goulart DR, de Moraes PH, Olate S, Barao VAR (2019). Comparative evaluation of different fixation techniques for sagittal split ramus osteotomy in 10 mm advancements. Part two: finite element analysis. J Craniomaxillofac Surg.

[16] Klein GBG, Mendes GCB, Junior PR, Viswanath A, Papageorge M (2017). Biomechanical evaluation of different osteosynthesis methods after mandibular sagittal split osteotomy in major advancements. Int J Oral Maxillofac Surg.

[17] Erkmen E, Şimşek B, Yücel E, Kurt A (2005). Comparison of different fixation methods following sagittal split ramus osteotomies using three-dimensional finite elements analysis: Part 1: advancement surgery-posterior loading. Int J Oral Maxillofac Surg.

[18] Borstlap WA, Stoelinga PJW, Hoppenreijs TJM, Van't Hof MA (2004). Stabilisation of sagittal split advancement osteotomies with miniplates: a prospective, multicentre study with two-year follow-up:Part I. Clinical parameters. Int J Oral Maxillofac Surg.

[19] Epker BN, Wessberg GA (1982). Mechanisms of early skeletal relapse following surgical advancement of the mandible. Br J Oral Surg.

[20] Pereira FL, Janson M, Sant'Ana E (2010). Hybrid fixation in the bilateral sagittal split osteotomy for lower jaw advancement. J Appl Oral Sci.

[21] Ureturk EU, Apaydin A (2018). Does fixation method affects temporomandibular joints after mandibular advancement?. J Craniomaxillofac Surg.

[22] Kobayashi T, Honma K, Hamamoto Y, Shingaki S, Nakajima T, Hanada K (2000). Effects of wire and miniplate fixation on mandibular stability and TMJ symptoms following orthognathic surgery. Clinical Orthodontics and Research.

[23] Ueki K, Okabe K, Marukawa K, Mukozawa A, Moroi A, Miyazaki M (2014). Skeletal stability after mandibular setback surgery:comparison between the hybrid technique for fixation and the conventional plate fixation using an absorbable plate and screws. J Craniomaxillofac Surg.

[24] Chuong CJ, Borotikar B, Schwartz-Dabney C, Sinn DP (2005). Mechanical characteristics of the mandible after bilateral sagittal split ramus osteotomy:comparing 2 different fixation techniques. J Oral Maxillofac Surg.

[25] Sato FRL, Asprino L, Noritomi PY, Da Silva JVL, De Moraes M (2012). Comparison of five different fixation techniques of sagittal split ramus osteotomy using three-dimensional finite elements analysis. Int J Oral Maxillofac Surg.

[26] Foley WL, Beckman TW (1992). In vitro comparison of screw versus plate fixation in the sagittal split osteotomy. Int J Adult Orthodon Orthognath Surg.

[27] Michelet FX, Deymes J, Dessus B (1973). Osteosynthesis with miniaturized screwed plates in maxillo-facial surgery. J Maxillofac Surg.

[28] Schwartz HC, Relle RJ (1996). Bicortical-monocortical fixation of the sagittal mandibular osteotomy. J Oral Maxillofac Surg.

